# The Association Between Professional Accounts on Social Networks Twitter and ResearchGate and the Number of Scientific Publications and Citations Among Anesthesia Researchers: Observational Study

**DOI:** 10.2196/29809

**Published:** 2021-10-15

**Authors:** Thomas Clavier, Emilie Occhiali, Zoé Demailly, Vincent Compère, Benoit Veber, Jean Selim, Emmanuel Besnier

**Affiliations:** 1 Department of Anesthesiology, Critical Care and Perioperative Medicine Rouen University Hospital Rouen France

**Keywords:** social network, anesthesia, publication, Twitter, ResearchGate, citation, social media, academic, researcher, bibliometrics, research output

## Abstract

**Background:**

Social networks are now essential tools for promoting research and researchers. However, there is no study investigating the link between presence or not on professional social networks and scientific publication or citation for a given researcher.

**Objective:**

The objective of this study was to study the link between professional presence on social networks and scientific publications/citations among anesthesia researchers.

**Methods:**

We included all the French full professors and associate professors of anesthesia. We analyzed their presence on the social networks Twitter (professional account with ≥1 tweet over the 6 previous months) and ResearchGate. We extracted their bibliometric parameters for the 2016-2020 period via the Web of Science Core Collection (Clarivate Analytics) database in the Science Citation Index-Expanded index.

**Results:**

A total of 162 researchers were analyzed; 42 (25.9%) had an active Twitter account and 110 (67.9%) a ResearchGate account. There was no difference between associate professors and full professors regarding active presence on Twitter (8/23 [35%] vs. 34/139 [24.5%], respectively; *P*=.31) or ResearchGate (15/23 [65%] vs. 95/139 [68.3%], respectively; *P*=.81). Researchers with an active Twitter account (median [IQR]) had more scientific publications (45 [28-61] vs. 26 [12-41]; *P*<.001), a higher h-index (12 [8-16] vs. 8 [5-11]; *P*<.001), a higher number of citations per publication (12.54 [9.65-21.8] vs. 10.63 [5.67-16.10]; *P*=.01), and a higher number of citations (563 [321-896] vs. 263 [105-484]; *P*<.001). Researchers with a ResearchGate account (median [IQR]) had more scientific publications (33 [17-47] vs. 26 [9-43]; *P*=.03) and a higher h-index (9 [6-13] vs. 8 [3-11]; *P*=.03). There was no difference between researchers with a ResearchGate account and those without it concerning the number of citations per publication and overall number of citations. In multivariate analysis including sex, academic status, and presence on social networks, the presence on Twitter was associated with the number of publications (β=20.2; *P*<.001), the number of citations (β=494.5; *P*<.001), and the h-index (β=4.5; *P*<.001).

**Conclusions:**

Among French anesthesia researchers, an active presence on Twitter is associated with higher scientific publication and citations.

## Introduction

In a globalized world, social networks have now taken a major place in the scientific field and are essential tools for promoting research and researchers (including the recruitment or the promotion of a researcher) [1-3]. Twitter is a microblogging service that allows its users to blog through short messages (ie, tweets). Each user can “retweet” a tweet from another user and broadcast it to his/her own followers, thus allowing fast dissemination of content. Because of its concise and synthetic nature, and given the possibility to follow specific thematic accounts, it is a social network used for professional purposes by many researchers and physicians [4]. ResearchGate is a social network reserved for researchers, used to promote their works and to connect with people working in the same field of research. It is thus a social network only dedicated to professional use for the international scientific community.

Medical journals use Twitter to increase their visibility within the scientific community. It is by far the most used social network to share publications because more than 20% of published articles receive at least one announcement on Twitter (compared with less than 5% of notifications on other nonprofessional social networks) [5]. It has been recently described that, among a selection of authors publishing in anesthesia journals, 24% have a Twitter account and 72% have a ResearchGate account [6]. Moreover, among French anesthesia, intensive care, and emergency medicine health care workers, 46% use social media to obtain information about medical actuality and 17% consult Twitter at least once a week [7]. This professional social network use should increase with the arrival of younger physicians because it was reported in a single-center study that 35% of medical students used Twitter for teaching purposes [8]. The link between social and usage metrics (altmetrics) and traditional bibliometric indicators is weak and variable, but Twitter’s altmetrics indicators seem to perform well in predicting the actual citation rate [9,10]. Twitter users tweet the articles they write and it is known that tweets can predict highly cited articles within the first days of an article publication [10-12]. Finally, recent randomized studies showed that, for a given journal, articles that benefited from exposure on Twitter were more cited than articles that were not tweeted [13,14]. However, there is no study investigating the link between presence or not on professional social networks and scientific publication or citation for a given researcher.

The objective of this study was to study the link between presence on social networks and scientific publication and citation among anesthesia researchers.

## Methods

### Study Design and Population

We used publicly available data; as a retrospective analysis that did not involve human participants (and in accordance with French laws), this study was exempt from institutional ethics board review [15].

We included all French physicians with an academic function of teaching and research in anesthesia (full professors and associate professors from the 48-01 subsection of the French National Council of Universities, Directory of Members for the year 2019).

### Objectives

The main objective of this work was to compare the scientific publication and citation of French anesthesia researchers according to the presence or absence of an active Twitter account. The secondary objectives were:

- to compare, in the same population, the scientific publication and citation according to the presence or absence on ResearchGate;
- to assess if the presence or absence from Twitter and ResearchGate was associated with scientific publication and citation of researchers.

### Data Extraction

To limit the impact of profile variations on social networks and publication citations in the bibliometric database, the entire data collection was carried out manually over 10 consecutive days in March 2021.

We analyzed the presence of included researchers on the social networks Twitter (professional account, that is, at least one follow of a profile related to anesthesia or intensive care medicine) and ResearchGate. The screening for finding social network accounts followed a step-by-step procedure:

- The first and last name were entered into the social network search engine. On Twitter, the author was searched on the account search tool and also on the “TOP” (most relevant Tweets for a given search) and “LATEST” (the most recently posted Tweets matching a given search) tabs;
- If no author was found after this first search, only the last name was used in association with the following keywords: “Dr”, “Pr”, “Anesthésie”, “Réanimation” (French keywords), “Anesthesia”, and “Intensive care”;
- If no author was still found, the first and last name were entered into the Google search engine with the keyword “Twitter” or “ResearchGate”;
- If several accounts were found for a given name, all accounts were manually analyzed in search of information on the account, to identify whether or not it was the researcher’s account (particularly through her/his hospital and/or academic affiliation).
- When an account was found, the following data were collected:
  For Twitter: existence of a professional Twitter profile (an account was considered active if it has published at least one professional tweet over the 6 months preceding the data collection date). For each active Twitter account, the recorded data were presence or not of a photograph, information concerning profession (academic or anesthetist), number of tweets, number of followers, and date of creation of the account (to determine the number of tweets and followers by month spent on Twitter);
  For ResearchGate, existence of an active ResearchGate profile (with a least one research work documented); if there is an existing profile, the following data were collected: presence or not of a photograph, number of followers, RG score (which is a measure of scientific reputation on ResearchGate), and Total Research Interest score (which is linked to the reading, citation, and recommendation of the researcher’s work on ResearchGate).
- Bibliometric parameters were extracted from the Web of Science Core Collection (Clarivate Analytics) database in the Science Citation Index-Expanded index. To limit the risk of errors due to homonyms in other research disciplines, we used a search algorithm focused on medical specialties that correspond to the fields of activity of anesthetists in France. Thus, the analysis focused on the publication of reviews, original articles, and editorials in medical journals over the period 2016-2020 on the advanced search tool of Web of Science with the following search formula:

(SU=CRITICAL CARE MEDICINE OR SU=ANESTHESIOLOGY OR SU=SURGERY OR SU=EMERGENCY MEDICINE OR SU=ALLERGY OR SU=CARDIAC & CARDIOVASCULAR SYSTEMS OR SU=CLINICAL NEUROLOGY OR SU=ENDOCRINOLOGY & METABOLISM OR SU=GASTROENTEROLOGY & HEPATOLOGY OR SU=HEMATOLOGY OR SU=IMMUNOLOGY OR SU=INFECTIOUS DISEASES OR SU=MEDICAL INFORMATICS OR SU=MEDICINE, GENERAL & INTERNAL OR SU=MEDICINE, RESEARCH & EXPERIMENTAL OR SU=MULTIDISCIPLINARY SCIENCES OR SU=NURSING OR SU=NUTRITION & DIETETICS OR SU=OBSTETRICS & GYNECOLOGY OR SU=PERIPHERAL VASCULAR DISEASE OR SU=PUBLIC, ENVIRONMENTAL & OCCUPATIONAL HEALTH OR SU=RESPIRATORY SYSTEM OR SU=TRANSPLANTATION OR SU=TOXICOLOGY) AND (PY=2016 OR PY=2017 OR PY=2018 OR PY=2019 OR PY=2020) AND (DT=ARTICLE OR DT=REVIEW OR DT=EDITORIAL MATERIAL) AND AU=“NAME OF THE AUTHOR, surname of the author”

For each researcher, the following parameters were recorded: number of publications, h-index, number of citations per publication, and overall number of citations.

### Statistical Analysis

The values are presented as n (%) for qualitative variables, and as median (IQR) for quantitative variables. The quantitative variables were compared using a Mann-Whitney *U* test. The qualitative variables were analyzed using a Fisher test. The Pearson correlation test was used to assess the strength of association between 2 quantitative variables. A multivariate analysis using a linear regression model was realized to identify whether the presence or absence from Twitter and ResearchGate was related to the number of citations, the h-index, and the number of publications. The multivariate analysis included the following variables: presence on Twitter, presence on ResearchGate, sex, and academic status (full professor or associate professor). All statistical tests were 2-sided and the .05 probability level was used to establish statistical significance. All statistics and graphs were produced using GraphPad PRISM software (version 9.1.2; GraphPad Software).

### Data Availability Statement

The raw data supporting the conclusions of this manuscript can be made available on request by the authors to any qualified researcher.

## Results

### Population Description

Of the 162 researchers analyzed (147 men and 15 women), 68 (42.0%) had a Twitter account, of which 42 (25.9%) were considered active, and 110 (67.9%) had a ResearchGate account. A total of 36 (22.2%) researchers had both an active Twitter account and a ResearchGate account. The characteristics of the Twitter and ResearchGate accounts identified are presented in Table 1.

There was no difference between associate professor and full professor regarding active presence on Twitter (8/23 (35%) vs. 34/139 (24.5%), respectively; *P*=.31) or on ResearchGate (15/23 (65%) vs. 95/139 (68.3%), respectively; *P*=.81).

**Table 1:** Characteristics of the Twitter and ResearchGate accounts.

Characteristics		Values^a^
**Active Twitter accounts (n=42)**		
	Specification of an anesthetist or academic function	25 (59.5)
	Photograph identifying the account owner	30 (71.4)
	Number of tweets/month	4.0 (1.5-11.0)
	Number of followers/month	4.4 (1.2-8.6)
**ResearchGate accounts (n=110)**		
	Photograph identifying the account owner	65 (59.1)
	RG score	39 (36-42)
	Total Research Interest score	1415 (779-2244)
	Number of followers	90 (50-168)

^a^Values are presented as n (%) for qualitative variables and as median (IQR) for quantitative variables.

### Scientific Publication and Citation According to the Presence or Absence of an Active Twitter Account

**Figure 1:**
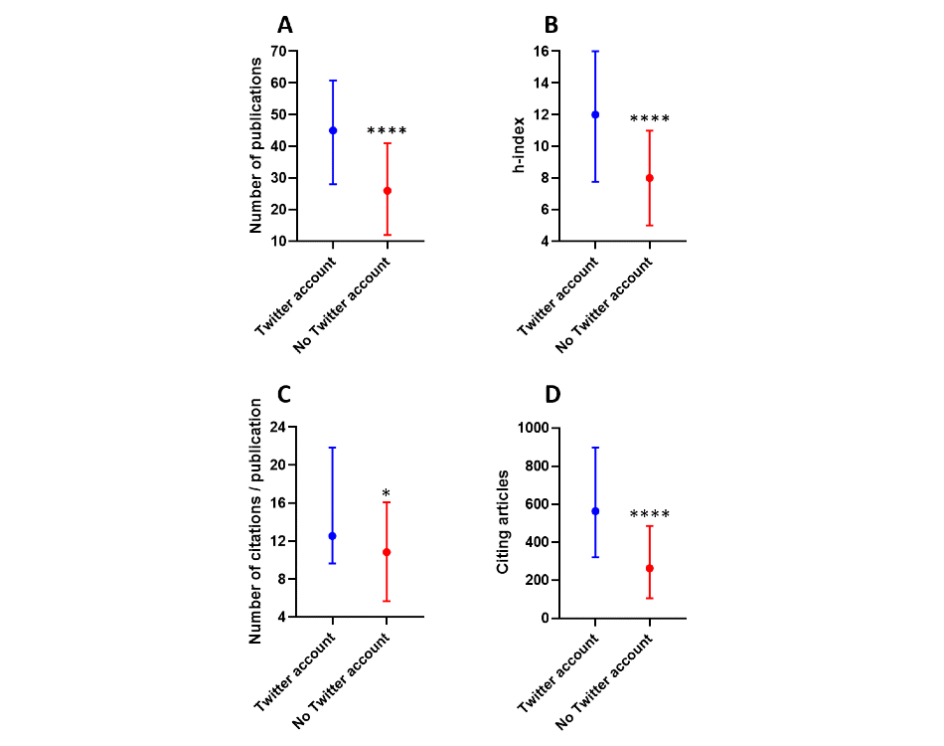
Number of scientific publications (A), h-index (B), number of citations per publication (C), and number of citing articles (D) over the period 2016-2020 among researchers with an active Twitter account. Data are presented as as median with interquartile range. *, *P*<.05; ****, *P*<.0001.

Over the period 2016-2020, researchers with an active Twitter account had more (median [IQR]) scientific publications (45 [28-61] vs. 26 [12-41]; *P*<.001; Figure 1A), a higher h-index (12 [8-16] vs. 8 [5-11]; *P*<.001; Figure 1B), a higher number of citations per publication (12.54 [9.65-21.8] vs. 10.63 [5.67-16.10]; *P*=.01; Figure 1C), and a higher number of citations (563 [321-896] vs. 263 [105-484]; *P*<.001; Figure 1D).

Among researchers with an active Twitter account, there was a correlation between the number of tweets/month and the number of followers/month (*r*=0.69; 95% CI 0.49-0.82; *P*<.001), the number of publications (*r*=0.37; 95% CI 0.08-0.61; *P*=.02), and the h-index (*r*=0.37; 95% CI 0.08-0.61; *P*=.02). There was also a correlation between the number of followers/month and the number of publications (*r*=0.43; 95% CI 0.14-0.64; *P*<.01) and the h-index (*r*=0.47; 95% CI 0.20-0.68; *P*<.01). There was no correlation between the number of tweets/month or the number of followers/month and the number of citations per publication or the overall number of citations.

### Scientific Publication and Citation According to the Presence or Absence of a ResearchGate Account

**Figure 2:**
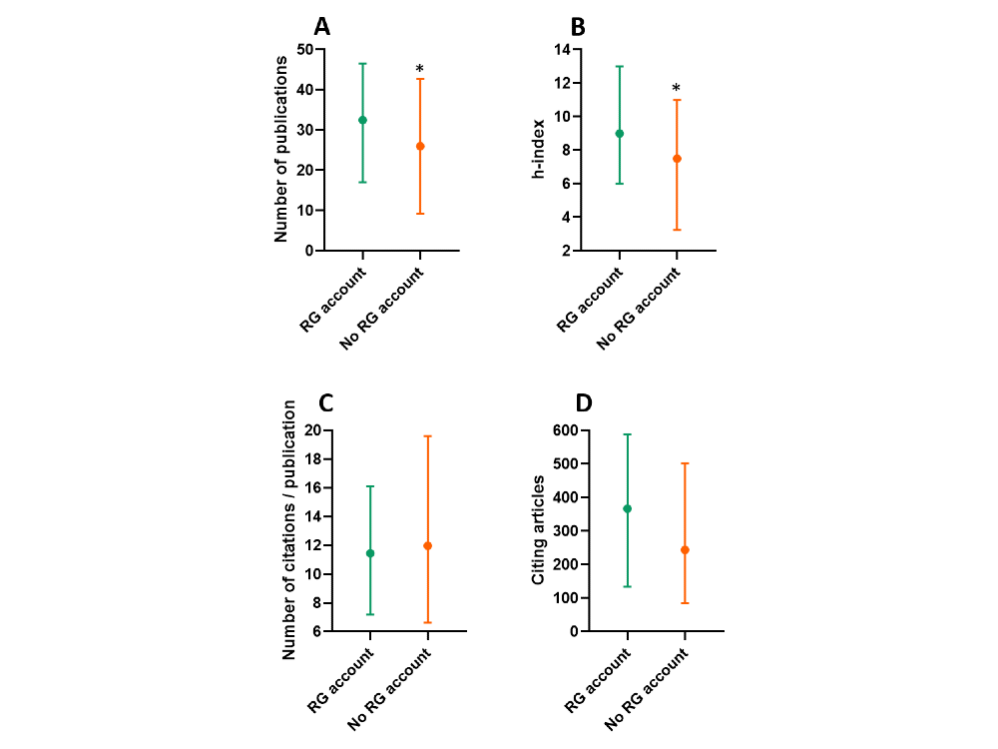
Number of scientific publications (A), h-index (B), number of citations per publication (C), and number of citing articles (D) over the period 2016-2020 among researchers with a ResearchGate (RG) account. Data are presented as as median with interquartile range. *, *P*<.05.

Over the period 2016-2020, researchers with a ResearchGate account had more (median [IQR]) scientific publications (33 [17-47] vs. 26 [9-43]; *P*=.03; Figure 2A) and a higher h-index (9 [6-13] vs. 8 [3-11]; *P*=.03; Figure 2B). There was no difference (median [IQR]) between researchers with a ResearchGate account and those without it concerning the number of citations per publication (11.45 [7.19-21.8] vs. 11.98 [6.62-19.62]; *P*=.67; Figure 2C) and the overall number of citations (367 [134-589] vs. 244 [85-502]; *P*=.17; Figure 2D).

Among researchers with a ResearchGate account, there was a correlation between the number of followers and the number of scientific publications (*r*=0.78; 95% CI 0.70-0.85; *P*<.001), the h-index (*r*=0.72; 95% CI 0.62-0.80; *P*<.001), the number of citations per publication (*r*=0.34; 95% CI 0.17-0.50; *P*<.001), and the overall number of citations (*r*=0.65; 95% CI 0.53-0.75; *P*<.001).

### Multivariate Analysis

In multivariate analysis, the presence on Twitter (but not on ResearchGate) was associated with the number of publications, the number of citations, and the h-index (Table 2).

**Table 2:** Multiple linear regression model to predict the number of citations, the h-index, and the number of publications.

Variable	Number of publications				Number of citations				h-index		
	β	95% CI	*P* value	β		95% CI	*P* value	β		95% CI	*P* value
Sex	11.8	–4.3 to 27.9	.15	236.8		–157.7 to 631.2	.24	2.2		–0.8 to 5.3	.15
Active presence on Twitter	20.2	9.2 to 31.2	<.001	494.5		224.8 to 764.1	<.001	4.5		2.4 to 6.6	<.001
Presence on ResearchGate	2.1	–8.2 to 12.3	.69	–158.8		–410.5 to 92.9	.21	0.45		–1.5 to 2.4	.65
Status	12.3	–1.9 to 26.6	.09	184.1		–165.9 to 534.2	.30	1.3		–1.5 to 4.0	.36

## Discussion

### Preliminary Findings

To our knowledge, we describe for the first time the participation rates and professional use patterns of social networks among all academics from a medical specialty of a given country. We also explore for the first time the link between researchers’ medical publication activity and their presence on social networks.

The 2 social networks analyzed in this work have different use in professional life. Twitter, which is not a network designed solely for professional use, allows subscribers to give their opinion, to follow some influencers, and also to exchange personal information. ResearchGate is reserved for researchers, and is used to promote their work and to connect with people working in the same field of research. Twitter is a network that requires active and frequent participation to disseminate information, whereas ResearchGate automatically imports publications from authors (who only has to validate them) and gives visibility to researchers with a high RG score, even if they are not very active on the network, allowing a more passive management of the account once it has been created. This may partially explain why the rate of researchers with a ResearchGate account is higher than that of researchers with an active Twitter account. Another likely explanation is that as ResearchGate is a social network specifically dedicated to research, it makes more sense for a researcher to be present there than to have a professional account on a mainstream network such as Twitter. The rates of use of professional social networks (Twitter: 42% (68/162) and ResearchGate: 67.9% (110/162)) are higher than those recently described among ENT surgeons, neurosurgeons, or pediatric orthopedists (Twitter: 2%-13% and ResearchGate: 23%-36%) [16-18]. The presence on Twitter of French anesthesia researchers also appears to be greater than that of nonacademic professionals from the same country working in the same field (17%) [7]. Nevertheless, the fact that we only included academic scientific authors in our analysis, versus physicians without academic activity in these other works, probably explains this difference. Thus, our rate of active Twitter users among researchers is closer to those recently described among health policy and health services researchers (30%) or among researchers publishing in anesthesia journals (between 22% and 25%) [6,19]. Similarly, the rate of use of ResearchGate appears similar to those described among academic researchers publishing in medical journals (between 45% and 70%) [6,20,21]. We can therefore assume that our data collection was relatively exhaustive in the study population. It is interesting to note that there was no difference in social networks between full professors and associate professors. This result should be interpreted with caution as there are few associate professors in our cohort and therefore probably a great lack of statistical power for this analysis. One explanation could be that, while associate professors belong to a generation more aware of social networks, full professors have had more time in their academic career to discover and use social networks for professional purposes. A significant proportion of Twitter and ResearchGate users do not have a photograph or description (or both in some cases) of their profession on their accounts. This may likely reduce their visibility on these social networks.

Among anesthesia researchers, an active presence on Twitter is associated with better bibliometric parameters, in terms of both number of publications and citations. The same trend is observed on ResearchGate. However, our study methodology does not allow to establish a causal link between scientific activity and presence on social networks. There are several possible explanations for the link we have identified. It is possible that very prolific researchers have the desire to disseminate their numerous publications and are therefore more inclined to use social networks. It is also possible that being on a professional social network allows to widely disseminate publications to the connected scientific community and thus have a greater chance of being read and cited. By analogy with the studies conducted on medical journals and showing the effectiveness of Twitter in increasing citations, it may seem logical that a researcher who posts his/her new publications on Twitter would also have more citations at an individual level [4,13,14]. It is also possible that sharing information on Twitter creates links and networks between some researchers who would be more likely to work together and thus increase their overall research activity and citation of each other. However, all these suggested explanations are only hypothetical and future studies seem necessary to explore a possible causal link between professional presence on social networks and scientific activity.

### Limitations

Despite interesting results, our study has several limitations. First, this work was limited to French anesthesia researchers on a 5-year period. Given the academic organization of the medical professions in France (which is centralized by the National Council of Universities), it is easy to have exhaustive access to the list of all academics in a given specialty. In other countries with a more decentralized academic organization (in particular, Anglo-Saxon countries), it is more complex to compile an exhaustive list of all academics in a country. In addition, social networks evolve very quickly (subscription/unsubscription, new followers/loss of followers, etc.), so it was essential to use data collected over a short period. However, because of the manual standardized procedure needed to detect all the accounts (which is time-consuming), the number of researchers that could be included was limited. Nevertheless, it seems necessary to confirm our observations in the academics of other countries. We chose the 2016-2020 period to analyze bibliometrics parameters because it corresponds to the period where social networks were started to be used in a massive way to promote research in France. It did not seem relevant for us to go further back in time, as the use of social networks was less common and the population studied would have been too disparate (old researchers who had retired or young researchers who had not yet started their academic activity). Second, even a standardized manual account search procedure has its flaws; some authors may use a pseudonym, a diminutive, or misspell their name when they register, etc. Third, some bibliometric parameters of ResearchGate such as RG score and total Research Interest score were not analyzed in our work. However, these scores are based on the number of publications and citations of researchers and have already been shown to be correlated with the h-index, the number of citations, the number of publication, and the academic level of registered researchers [9,18,22,23]. Thus, it appeared futile to search for correlation between these scores and the bibliometric parameters found on Web of Sciences. Moreover, ResearchGate metric parameters are controversial to assess research output of researchers, especially the RG score, which is built from both activity related to asking and answering questions on the website and not just from publication metrics [24]. We therefore did not consider it relevant to include these scores in our analysis. Fourth, we did not analyze other famous social networks (eg, Instagram, Facebook), but because these networks are mainly dedicated to recreational use and are rarely used by physicians in a professional context, it did not seem relevant for us to include them in a study dedicated to professional impact of social networks. We also did not include LinkedIn in our analysis. Although it is a professional social network, it gives little access to profile data: no quantification of the number of posts and no precise quantification of the number of relationships beyond 500 relationships. It therefore seemed difficult to have relevant markers for this network.

### Conclusion

Among French anesthesia researchers, an active presence on Twitter is associated with higher scientific publication and citations. Future studies could explore a possible causal link between these parameters.
